# Glycine decarboxylase as a novel regulator of N-methyl-D-aspartate receptor function: Implications for pathophysiology of schizophrenia

**DOI:** 10.4103/NRR.NRR-D-25-00318

**Published:** 2025-11-25

**Authors:** Maltesh Kambali, Uwe Rudolph

**Affiliations:** Department of Comparative Biosciences, College of Veterinary Medicine, University of Illinois Urbana-Champaign, Urbana, IL, USA; Neuroscience Program, University of Illinois Urbana-Champaign, Urbana, IL, USA; Carl R. Woese Institute for Genomic Biology, University of Illinois Urbana-Champaign, Urbana, IL, USA

**Glutamate receptors and schizophrenia:** Schizophrenia is a chronic mental disorder affecting approximately 1% of the global population, with 70%–80% heritability. It has a multifactorial etiology involving both environmental factors and a complex polygenic genetic architecture. Over the last two decades, large-scale genome-wide approaches revealed contributions of common variants with individually small effect sizes and of rare copy number variants with a large effect size.

N-methyl-D-aspartate receptor (NMDAR) hypofunction has been implicated as a central mechanism in the pathophysiology of schizophrenia (Coyle et al., 2020). Although exonic mutations affecting key neurodevelopmental pathways occur infrequently, a meta-analysis identified deleterious coding variants in 10 genes conferring substantial risk for schizophrenia, including *GRIN2A*, the gene encoding the NMDAR subunit 2A and *GRIA3*, the gene encoding the AMPA (α-amino-3-hydroxy-5-methyl-4-isoxazolepropionic acid) receptor subunit 3, pointing to a role of ionotropic glutamate receptors in the pathophysiology of schizophrenia (Singh et al., 2022). Genome-wide association studies identified 270 loci linked to the disorder, implicating 130 risk genes (Coyle et al., 2020). Approximately 30% of these genes encode proteins that localize to glutamatergic synapses, particularly within the pre- and postsynaptic compartments (Coyle et al., 2020). Glycine and D-serine are required co-agonists of the NMDAR. Serine racemase knockout mice that do not convert L-serine to D-serine have been reported to display schizophrenia-like phenotypes (Coyle et al., 2020). This perspective will primarily on the role of glycine decarboxylase (GLDC), the rate-limiting enzyme of the glycine cleavage system (GCS), which in the brain is expressed primarily in astrocytes, in modulating NMDAR-dependent cellular physiological responses, biochemical pathways, and behavioral functions. Loss-of-function mutations in *GLDC* and other GCS components result in non-ketotic hyperglycemia and neural tube defects. Here, we will review recent evidence suggesting that an increased copy number of *GLDC* results in NMDAR hypofunction and contributes to schizophrenia-like phenotypes, and compare NMDAR hypofunction mouse models reducing the availability of the co-agonists D-serine and glycine.

**Figure 1:**
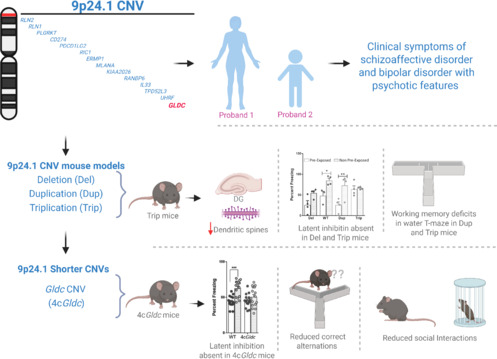
9p24.1 CNVs in human patients and chromosome-engineered mouse models. The 9p24.1 CNV spanning 14 genes was identified in two patients (proband 1 and proband 2) presenting with clinical symptoms of psychosis (schizoaffective disorder and bipolar disorder with psychotic features). The *GLDC* gene, highlighted in red color, was the only gene triplicated, and the other genes were fully duplicated in patients. To study the pathophysiological significance of the CNV, mice with the full 9p24.1 CNV [deletion, duplication, and triplication (i.e., homozygous duplication)] were generated with gene targeting, *trans*-allelic recombination, and selective breeding. Further, to identify which genes contribute to the observed phenotypes, using CRISPR/Cas-9 and the techniques mentioned above, CNV mice with 4 copies of Gldc (4c*Gldc* mice) generated. The observed phenotypes segregated completely with increased copy number of the *Gldc* gene. Created with BioRender.com. The latent inhibition graphs were reprinted with permission from Kambali et al. (2025). CNV: Copy number variant; GLDC: glycine decarboxylase.

**From a 9p24.1 copy number variant to NMDAR dysfunction:** A rare 9p24.1 copy number variant (CNV), a 1.8 Mb duplication/triplication involving 14 genes, including glycine decarboxylase (GLDC), was found in a family where both mother (Proband 1) and son (Proband 2) carry a small supernumerary marker chromosome derived from chromosome 9, which segregates with psychotic illness (Bodkin et al., 2019; **Figure 1**). The potential pathogenic significance of this CNV for the development of schizophrenia-associated phenotypes is unclear. However, given the established role of GLDC in glycine catabolism and the fact that glycine is a co-agonist at the NMDAR, we hypothesized that GLDC overexpression would negatively impact NMDAR function by reducing glycine availability, which might lead to schizophrenia-like behavioral deficits. We indeed found that mice mimicking the 9p24.1 CNV display schizophrenia-like phenotypes (Kambali et al., 2025; **Figure 1**). To identify the gene(s) driving the observed phenotypes, mouse models with an increased copy number of either the *Gldc* gene (4c*Gldc* mice, homozygous for the duplication allele) or of all other genes included in the 9p24.1 CNV (4c*Rln1-Uhrf2* mice) were generated (**Figure 1**).

**Figure 2:**
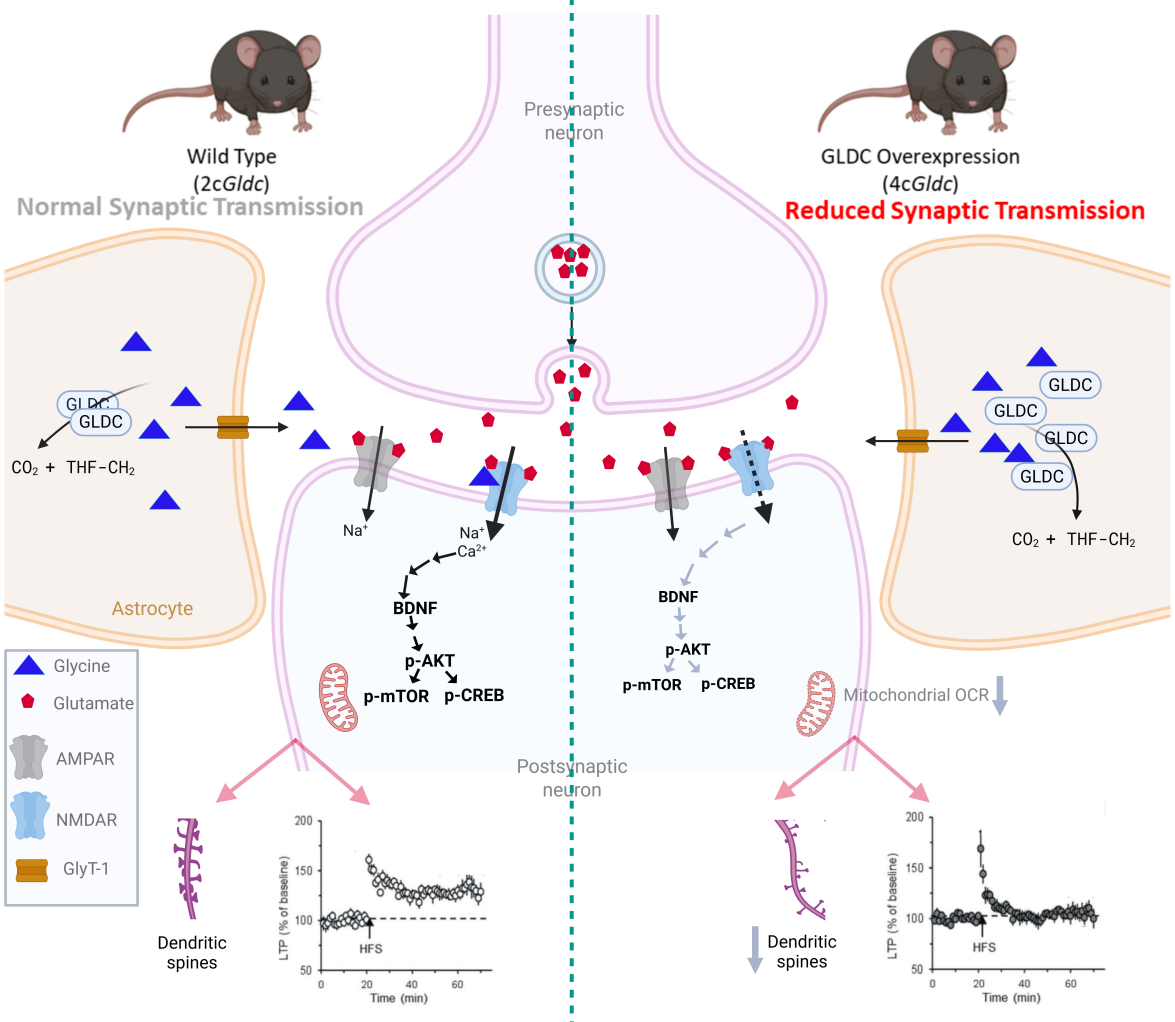
Key pathophysiological alterations in the dentate gyrus of 4c *Gldc* mice (right) compared to wild-type 2c *Gldc* mice (left). In 4c*Gldc* mice, the extracellular glycine levels (blue triangles), as determined using the fluorescent glycine FRET sensor GlyFS, are reduced in the dentate gyrus. The glutamate levels (red pentagons) are presumably unaltered. However, elevated GLDC expression in astrocytes of 4c*Gldc* mice leads to reduced availability of glycine in the synapse. In 4c*Gldc* mice, there is thus less extracellular glycine that can bind to NMDARs. This leads to diminished activation (grey arrows) of postsynaptic neurons and activity-dependent downstream pathways involving BDNF, AKT, mTOR and CREB, reduced dendritic spine density, and impaired LTP. In addition, mitochondrial function was impaired in 4c*Gldc* mice, as evidenced by a reduction of the mitochondrial OCR in the Seahorse assay. For further details of pathophysiological mechanisms see text. Created with BioRender.com. The LTP panels were reprinted with permission from Kambali et al. (2025). AMPAR: Alpha-amino-3-hydroxy-5-methyl-4-isoxazolepropionic acid receptor; BDNF: brain-derived neurotropic factor; GLDC: glycine decarboxylase; GlyT-1: glycine transporter 1; HFS: high-frequency stimulation; LTP: long-term potentiation; NMDAR: N-methyl-D-aspartate receptor; OCR: oxygen consumption rate; p-AKT: phosphorylated Akt; p-CREB: phosphorylated cyclic AMP response element-binding protein; p-mTOR: phosphorylated mammalian target of rapamycin; THF-CH_2_: N5,N10-methylene-tetrahydrofolate.

**Region-specific impact of GLDC overexpression on NMDAR function:** In 4c*Gldc* mice, extracellular glycine concentrations are selectively reduced in dentate gyrus (DG) (**Figure 2**) but not in the hippocampal subfield CA1, as detected using the optical glycine FRET sensor GlyFS (Kambali et al., 2025), resulting in impaired NMDAR-dependent activity, specifically reduced long-term potentiation (LTP) in DG, without affecting LTP in CA1 (**Figure 2**). These observations underscore the reliance of NMDARs in the DG on a pool of extracellular glycine regulated by *GLDC*, consistent with previous work showing that glycine is the predominant co-agonist in DG and D-serine the predominant co-agonist in CA1 (Le Bail et al., 2015). More specifically, the novel findings establish that GLDC negatively regulates NMDAR function. LTP deficits have also been described in other mouse models for schizophrenia with NMDAR hypofunction. In contrast to 4c*Gldc* mice, serine racemase knockout (SRKO) mice display LTP deficits in both CA1 and DG (Balu et al., 2013; Coyle et al., 2020). *Grin2a* heterozygous and homozygous knockout mice also show LTP impairments in CA1 and DG. *Grin2a* heterozygous knockout mice exhibit hippocampal hyperactivity and prefrontal cortex hypoactivity, while *Grin2a* homozygous knockout mice exhibit hyperlocomotion and spatial pattern separation deficits, which is relevant as pattern separation is a DG-dependent task. The results obtained with the 4c*Gldc* mice indicate that an LTP deficit in the DG may be sufficient for the induction of schizophrenia-like phenotypes (described below). Support for the central role of DG activity for psychosis also comes from recent work showing that chemogenetic inhibition of DG granule cells for 21 days in adolescent (6-week-old) mice results in hippocampal hyperactivity and psychosis-like behavioral outcomes (Scott et al., 2025).

**Neurobiology of GLDC overexpression:** Cultured primary astrocytes derived from mice with four copies of the entire 9p24.1 CNV locus exhibited increased expression of GLDC, phosphoglycerate dehydrogenase (PHGDH), and serine racemase (Li et al., 2018). Biochemical analysis of the conditioned medium revealed decreased glycine, increased D-serine, and reduced L-serine levels (Li et al., 2018). This metabolic reconfiguration may reflect an elevated flux through the serine synthesis pathway, possibly compensating for the reduction of glycine levels.

Biochemical analyses of hippocampal synaptoneurosomal fractions from 4c*Gldc* mice revealed significant downregulation of brain-derived neurotrophic factor (BDNF), a central regulator of synaptic plasticity, and of BNDF-dependent signaling, with strikingly similar results in SRKO mice and 4c*Gldc* mice (Balu et al., 2013; Kambali et al., 2025). BDNF mRNA and protein levels were markedly reduced, accompanied by impaired activation of its downstream signaling pathways (**Figure 2**). Notably, phosphorylation of Akt at Ser473 and Thr308, as well as phosphorylation of mammalian target of rapamycin (mTOR) at Ser2448, was significantly diminished, despite unaltered total Akt and mTOR protein levels. Although P70S6K, a downstream target of mTOR involved in ribosomal biogenesis and protein synthesis, exhibited no changes in phosphorylation in 4c*Gldc* mice (as opposed to a reduction in SRKO mice), phosphorylated CREB (p-CREB), a transcription factor essential for activity-dependent gene expression and synaptic remodeling, was significantly reduced, despite stable total CREB levels (**Figure 2**). These findings suggest that overexpression of GLDC disrupts BDNF-Akt-mTOR and BDNF-Akt-CREB signaling and establishes a potential mechanistic link between overexpression of GLDC and synaptic dysfunction, reflecting a molecular mechanism thought to play a role in schizophrenia-related pathophysiology. As mentioned before, SRKO mice also exhibit impairments in BDNF signaling and activation of Akt/mTOR-mediated pathways (Balu et al., 2013).

Transcriptomic analysis of 9p24.1 CNV mice revealed that in the hippocampus of mice with a triplication of all 9p24.1 genes, several KEGG pathways are downregulated, including long-term potentiation, chemokine signaling pathways, dopaminergic synapse, and GABAergic synapse. Notably, the expression of two genes that have been associated with schizophrenia by genome-wide association studies was significantly altered in the hippocampus: miR-137, a key regulator of synaptic plasticity and cognitive function, was upregulated in the triplication genotype, and ARL3, a critical mediator of intracellular trafficking, was downregulated in both duplication and triplication genotypes. Moreover, differentially expressed genes with rare coding variants linked to autism and neurodevelopmental disorders were enriched (Kambali et al., 2025). These findings indicate that an increase in the copy number of the 9p24.1 genes, which in itself is not associated with psychiatric disorders at a genome-wide level, also modulates pathways that have been found to be linked to schizophrenia, autism, and other neurodevelopmental disorders. Taken together with findings in the SRKO mice (Coyle et al., 2020), these results show that glutamatergic hypofunction is sufficient to elicit deficits in multiple other pathways, including dopaminergic pathway.

Both 4c*Gldc* and SRKO mice display reduced dendritic spine density, a hallmark of schizophrenia (Coyle et al., 2020). The microRNA miR-132, known to regulate synaptic plasticity and spine formation, is downregulated in both models. Moreover, elevated expression of miR-137, another schizophrenia risk gene known to reduce spine density, may contribute to synaptic alterations in the 4c*Gldc* mouse model. These findings suggest that genetic modifications resulting in reduced levels of the NMDAR co-agonists glycine or D-serine can modulate synaptic structure via microRNA-mediated pathways. As the behavioral analysis of SRKO and 4c*Gldc* mice was only partially overlapping, comparisons are limited (Balu et al., 2013; Kambali et al., 2025). The 4c*Gldc* mouse displayed a weak prepulse inhibition of acoustic startle deficit, while the SRKO mice did not. Moreover, the 4c*Gldc* mice displayed a startle habituation deficit, while the SRKO mice did not. In contextual fear conditioning, 4c*Gldc* mice displayed increased freezing, while SRKO mice displayed reduced freezing. Likewise, in trace fear conditioning the 4c*Gldc* mice display increased freezing, while the SRKO mice displayed reduced freezing. Several paradigms in which the 4c*Gldc* mice showed deficits (**Figure 1**), i.e., latent inhibition to conditioned freezing, working memory (in Y maze and water T maze), and social interaction (in the three-chamber test), have apparently not been performed in SRKO mice. These comparisons indicate that while reduced levels of glycine in the 4c*Gldc* mice and reduced levels of D-serine in the SRKO mice have relatively similar biochemical and neurophysiological consequences, they have distinct behavioral consequences, highlighting the distinct yet partially overlapping roles of glycine and D-serine in NMDAR modulation and schizophrenia pathophysiology. Interestingly, some behavioral deficits in the SRKO mice can be rescued with administration of D-serine (Balu et al., 2013), and administration of glycine reverses behavioral deficits in 4c*Gldc* mice (manuscript in revision). In a precision medicine approach, in patients with the 9p24.1 CNV augmentation of clozapine with glycine or D-cycloserine improved psychotic and mood symptoms (Bodkin et al., 2019). GLDC itself is likely not a suitable drug target as it is expressed in many tissues outside of the brain.

**GLDC overexpression induces mitochondrial dysfunction:** Cerebral organoids derived from patients with schizophrenia were found to display reduced basal oxygen consumption, adenosine triphosphate production, and a proton leak, highlighting mitochondrial dysfunction in schizophrenia. When mitochondrial respiration was measured in hippocampal slices of 4c*Gldc* mice, the basal oxygen consumption rate and the FCCP (carbonyl cyanide p-trifluoromethoxyphenylhydrazone)-stimulated maximal respiration were significantly reduced in DG but not in CA1 (Kambali et al., 2025). This is in line with the observations mentioned above that in these mice, extracellular glycine and LTP are reduced in DG (**Figure 2**) but not in CA1. Notably, the basal respiration was lower in DG, a region in which glycine is the major co-agonist, than in CA1, a region in which D-serine is the major co-agonist (Kambali et al., 2025), indicating that mitochondrial respiration deficits may be considered to be a downstream effect of impaired excitatory neurotransmission. Recent evidence indicates that targeting mitochondrial respiration may be a useful therapeutic approach. Metformin, an antidiabetic drug that inhibits mitochondrial complex I and thus may increase mitochondrial biogenesis, reversed schizophrenia-like symptoms in a MK801-induced rat model of schizophrenia, attenuating sensorimotor gating deficits (i.e., prepulse inhibition impairments), reversing hyperlocomotion, and rescuing working memory impairments (Chen et al., 2021). These behavioral effects were associated with the normalization of dysregulated intracellular signaling, specifically Akt and GSK3β phosphorylation in the frontal cortex (Chen et al., 2021). A complementary study found that 24 weeks of metformin treatment improved cognitive function in patients with schizophrenia, specifically working memory and verbal learning, by modulating TCA cycle metabolites and enhancing hippocampal functional connectivity (Chen et al., 2021; Shao et al., 2023). Thus, targeting mitochondrial respiration by inducing mitochondrial biogenesis and/or improving mitochondrial function may be a clinically relevant approach, and the 4c*Gldc* mice are a suitable model for preclinical studies testing novel therapeutic strategies targeting mitochondria.

**Conclusions:** At a fundamental level, recent studies of the 9p24.1 CNV add to our knowledge of how synaptic activity is modulated, establishing GLDC as a negative regulator of NMDAR-mediated neurotransmission in physiological and pathophysiological contexts. They suggest that a reduction of extracellular glycine levels in DG may contribute to neurodevelopmental and psychiatric disorders. Intriguingly, there are notable functional parallels and differences between deficiencies of the NMDAR co-agonists glycine and D-serine. Furthermore, NMDA receptor hypofunction induced by reduced glycine levels in the DG is sufficient to induce mitochondrial respiration deficits, which may be closely linked to or even underlie many biochemical and behavioral deficits observed and thus may represent a target for the development of novel pharmacological strategies.
